# Composition, Physicochemical and Sensorial Properties of Commercial Plant-Based Yogurts

**DOI:** 10.3390/foods9030252

**Published:** 2020-02-26

**Authors:** Nadia Grasso, Loreto Alonso-Miravalles, James A. O’Mahony

**Affiliations:** School of Food and Nutritional Sciences, University College Cork, T12 Y337 Cork, Ireland; 117107686@umail.ucc.ie (N.G.); 116221127@umail.ucc.ie (L.A.-M.)

**Keywords:** plant-based food, dairy-free yogurt, physicochemical properties, rheology, texture

## Abstract

The aim of this study was to determine the key physicochemical, sensory and quality attributes of plant-based yogurts made from soy, coconut, cashew, almond and hemp, including a dairy benchmark yogurt. The soy, coconut and cashew-based yogurts showed textural parameters comparable to the dairy yogurt, with firmness values of 0.46, 0.44, 0.51 and 0.36 N, respectively. Rheological analysis showed that one of the soy-based yogurts was similar to the dairy yogurt in terms of apparent viscosity, in addition to water-holding capacity (82.8% and 75.7%, respectively). Other plant-based yogurts, e.g., hemp, showed different rheological and textural parameters to the other plant-based products, relating this to the agar and rice starch components of the hemp formulation. The sensory analysis demonstrated that some plant-based yogurts were similarly appreciated to dairy-based products. This was due mainly to the presence of specific hydrocolloids, sweeteners and flavours in the formulations; for example, the acceptability of the soy- and dairy-based yogurts were identical (5.95). The results obtained in this study allowed identification of key quality attributes of plant-based yogurt products and highlighted relationships between such attributes and formulation, which can be exploited in future product development.

## 1. Introduction

The increase of global food demand will lead to a substantial increase in overall food production by 2050 [1] due to the estimated growth of the global population by more than one billion people over the next 13 years, reaching 9.8 billion by 2050 [2]. Over the last 50 years, the daily intake of protein has increased in high-income countries, particularly coming from meat, eggs, milk and dairy products, increasing from 39 to 52 g per capita between 1961 and 2011. The Food and Agriculture Organization estimates for 2030 and 2050 daily protein intakes of 54 and 57 g per person, respectively [3]. Moreover, the type of food demanded by the consumer has changed, is still changing, and will continue to change in the coming years due to the higher standards of living in developing countries and urbanisation, eventually leading to an increase in animal food production if existing trends continue uninterrupted [4,5]. On the other hand, increased consumer awareness about the impacts of food production and consumption on the environment and health is contributing to a decrease in the demand of animal-derived food products in developed countries [6]. Therefore, the focus of many food producers and researchers is to address the present consumer demands and environmental concerns by creating healthy and sustainable alternative food products [7].

The consumption of plant protein is increasing in Europe, and this is reflected in the annual growth rates of 14% and 11% for meat and dairy alternatives, respectively [8]. Plant-based yogurts represent an important segment among the dairy-free alternatives, meeting the needs of many consumers, such as those with dairy allergies and ethical concerns [9]. In 2016, the number of new product launches of plant-based yogurts was 20% higher compared with 2015 [10]. Plant-based yogurts are generally made by fermenting aqueous extracts obtained from different raw materials (e.g., legumes, oil seeds, cereals or pseudocereals), with such extracts having appearance and consistency similar to cow’s milk resulting from the breakdown and homogenisation of these materials [7,11,12]. Among the plant-based sources used for yogurt production, soybean has been especially popular in recent decades because of its protein quantity, quality and functional properties [13,14]. Furthermore, soymilk has been shown to be a good substrate for the growth of lactic acid bacteria commonly used during yogurt fermentation [15]. However, while soy has been the most widely used substrate in the production of plant-based yogurts, nowadays other substrates are emerging, such as those derived from coconut [10].

The major challenges faced by producers of plant-based yogurts are associated with the appearance and texture properties as such products generally have textural issues caused by phase separation. On acidification of such plant-based systems, destabilisation of the proteins results in the formation of a non-continuous, weak gel, resulting in serum separation [16]. For this reason, hydrocolloids are usually employed in the formulation of plant-based yogurts in order to stabilise the particles in suspension, contribute to structure formation and help with the imitation of the characteristics of a dairy-based yogurt [17]. Combinations of gelling agents (e.g., natural gums, proteins, starches, pectin and agar) are often used in the food industry to provide gel-type food products (e.g., yogurts and puddings) with acceptable texture [18]. However, little research has been published on the rheological, sensory and related quality attributes of these types of food products.

The aim of this study was to determine the physicochemical, rheological and sensory properties of six commercial plant-based yogurts in order to develop an understanding of how the different formulations can influence key quality attributes. In addition, a dairy-based yogurt was included as a benchmark with the aim of understanding which plant-based formulation gave properties most similar to the benchmark. This study will provide much needed knowledge about the formulation of plant-based yogurts and the effects of different ingredients (i.e., plant-based substrate and choice of hydrocolloid) on texture and mouthfeel, assisting with development of next-generation plant-based yogurt systems.

## 2. Materials and Methods 

### 2.1. Yogurt Products

Six commercial plant-based yogurts were analysed in this study, in addition to a dairy yogurt as a benchmark. The plant-based yogurts were made from soy (1 and 2), coconut, cashew, almond and hemp. Products were purchased from a number of local commercial retail outlets (Quay Co-op, Tesco and Lidl, Cork, Ireland). The nutritional composition and details of ingredients used in the product formulations are reported in Table 1 and Table 2, respectively. All samples were declared as fermented yogurts and were of plain type (i.e., not flavoured).

### 2.2. pH and Lactic Acid Content 

The pH of the commercial yogurts was measured with a pH meter (Mettler Toledo, Greifensee, Switzerland) at 20 °C. D- and L-Lactic acids were determined using an enzymatic reagent kit (D-/L-Lactic acid kit, K-DLATE 07/14, Megazyme, Wicklow, Ireland).

### 2.3. Total Titratable Acidity 

Titratable acidity was determined by neutralising the acid present in 10 g of the commercial yogurt samples using 0.11 N NaOH solution. The titration was performed using 10 drops of phenolphthalein as indicator until a pink endpoint was reached. The quantity of NaOH used to neutralise the solutions was divided by 10 in order to obtain the titratable acidity.

### 2.4. Colour 

The colour of each sample was measured using a chromameter CR-400 (Konica Minolta Sensing, Inc, Osaka, Japan) using CIELAB coordinates (L*, a*, b*). The chromameter was calibrated before the measurement using a white tile. In the CIELAB colour space system, L* value corresponds to the brightness and the values can vary between 0 (black) and 100 (white), a* value measures degree of redness (positive values) or greenness (negative values), and b* value measures degree of yellowness (positive values) or blueness (negative values).

### 2.5. Water Holding Capacity 

The method described by Silva and O’Mahony [19] was followed for analysis of water holding capacity. In brief, 20 g of commercial yogurt was placed in 50 mL tubes (Thermo Fisher Scientific, Waltham, MA, USA) and centrifuged at 640× *g* for 20 min at 4 °C in a Sorvall RC 5C Plus centrifuge, equipped with a Sorvall SS-34 rotor (Du Pont Instruments, Wilmington, DE, USA), after which the supernatant was collected and weighed. Water holding capacity (*WHC*) was calculated according to Equation (1):

(1) $$WHC\;\left(\%\right)=\left(1-\frac{weight\;of\;supernatant}{weight\;of\;sample}\right)\times \;100$$

### 2.6. Rheological Analysis 

#### 2.6.1. Uniaxial Compression Testing

Textural properties of commercial yogurts were measured using the back extrusion method as described by Silva and O’Mahony [19]. The test was performed using a Texture Analyser TAXT2i (Stable Micro Systems Ltd., Godalming, Surrey, UK), equipped with a 25 kg load cell and an extrusion disc (Ø = 35 mm), operating at a fixed test speed of 1.0 mm s^−1^, to a depth of 25 mm. Samples (100 g) were weighed and stored overnight at 4 °C in beakers of 150 mL to allow the samples to equilibrate. The force-time curves were analysed using Texture Expert Exceed (Stable Micro Systems Ltd., Godalming, Surrey, UK). After the calibration, and immediately after removal from storage at 4 °C, firmness, which is the maximum positive force in compression, consistency, which is the positive area of the curve, cohesiveness, which is the maximum negative force of the curve, and the viscosity index, which is the negative area of the curve, were measured.

#### 2.6.2. Viscosity 

Apparent viscosity (η) of commercial yogurts was measured at 20 °C using a rotational viscometer (Haake RotoVisco 1 Rotational Viscometer, Thermo Fisher Scientific, Waltham, MA, USA) equipped with a cup and bob geometry (Z41, Z43, Thermo Fisher Scientific, Waltham, MA, USA). After calibration of the instrument, samples (20 g) were loaded into the cup and an increasing shear rate ($\dot{\gamma }$) from 0 to 200 s^−1^ was applied for 180 s, then a steady shear rate ($\dot{\gamma }$) of 200 s^−1^ was applied for 120 s, after which a decreasing shear rate ($\dot{\gamma }$) from 200 to 0 s^−1^ was applied for 180 s. Yield stress (*τ*_0_), consistency coefficient (*K*) and the flow behaviour index (*n*) were calculated according to the equation of the Herschel-Bulkley model (Equation (2)):

(2) $$\tau =\tau_{0}+K\dot{\gamma^{n}}$$

### 2.7. Sensory Analysis

A hedonic test was carried out by a consumer panel (25 panelists, two sessions) consisting of students and staff recruited within University College Cork, Ireland. Samples (10 g) were served at 4 °C in a randomised order in transparent plastic cups with three-digit codes and rated using a 100 mm line scale (appearance, odour, flavour, texture and overall acceptability), where 0 corresponded to “extremely dislike” and 10 to “extremely like”. After finalising the sensory evaluation of the yogurts, the panelists completed a questionnaire on yogurt (dairy and/or plant-based) consumption habits and a food neophobia scale (FNS) questionnaire [20]. The sensory analysis was conducted without inclusion of the hemp sample due to challenges with availability of commercial samples.

### 2.8. Statistical Data Analysis 

All analyses were performed in triplicate except for the total titratable acidity and the lactic acid analyses, which were performed in duplicate. The data for all parameters measured were initially examined for normality using SPSS version 25 (SPSS Inc., Chicago, IL, USA). The Kruskal–Wallis non-parametric test, alternative to one-way analysis of variance, was used to make conclusions about the equality of medians between the samples, which were not normally distributed. The post-hoc Dunn-Bonferroni test was used to compare the samples in pairs, defining the significant differences between samples.

## 3. Results and Discussion

### 3.1. Chemical Composition

The nutritional composition of the yogurts is provided in Table 1. The protein content varied from 0.6 to 4.6 g/100 g for plant-based yogurts, with the dairy yogurt having the highest protein content of 5.1 g/100 g, comparable with the values for commercial products reported previously [21,22] and the coconut and hemp yogurts having the same low protein content of 0.6 g/100 g. Furthermore, the almond yogurt had the highest (7.9 g/100 g) and the dairy yogurt the lowest (1.5 g/100 g) fat content. The saturated fat content was considerably higher for the coconut yogurt (4.2 g/g fat) than the other yogurts (0.2–1.0 g/g fat). The high fat content in some plant-based yogurts (i.e., almond and coconut) resulted in higher caloric density (79 and 97 kcal, respectively) compared to the other yogurts (38–70 kcal). The coconut yogurt had the highest carbohydrate and sugar contents (8.0 and 4.3 g/100 g, respectively). The soy-2 and the dairy yogurts had the simplest formulations with no added hydrocolloids (Table 2). On the other hand, additives such as starch, pectin and agar were used in the formulation of all the other plant-based yogurts, with four of them containing combinations of hydrocolloids (e.g., rice starch and agar for the hemp-based yogurt, tapioca starch and carob gum for the cashew and almond-based yogurts). Flavourings were also included in the soy-1 and coconut-based yogurts.

### 3.2. Total Titratable Acidity, pH and Lactic Acid

The pH values of all the yogurts analysed ranged from 3.99 to 4.56 (Table 3); of note is the lower pH of the hemp sample (3.99) compared to the other yogurts (4.00–4.56). These pH data can be explained by the ingredients used in the yogurts, such as acidity regulators (e.g., citric acid and sodium citrate) that generate a pH that is preferable or essential for certain food processes, such as yogurt production [23]; moreover, the same authors claimed that specific ranges of pH are preferred since they influence the action of some gelling agents. The total titratable acidity (TTA) value was higher for the dairy yogurt (1.38 mL NaOH/g), in agreement with the values reported by Laye et al. [24] for commercial samples, than for the plant-based yogurts (0.12–0.78 mL NaOH/g), with the lowest TTA measured in the hemp yogurt (0.12 mL NaOH/g). A similar trend was observed for the L-lactic acid content, with the dairy yogurt having the highest value (1.11 g/100 g), implying that the bacterial fermentation achieved was more effective with a lactose substrate (i.e., dairy yogurt) than with the carbohydrate substrates in the plant-based yogurts. The type of lactic acid isomer produced (D- or L-) during the fermentation of the yogurt is species-specific [25], consequently the cultures used for the production of all the yogurts were L-lactic acid producers as concentrations of L-lactic acid were considerably higher than those for D-lactic acid for all samples.

### 3.3. Colour

Colour is an important consideration for the acceptability of food products by consumers and the CIELAB system colour results (L*, a*, b*) for the samples are shown in Table 4. The dairy product had the highest L* and the most negative a* values. The brightness of yogurts is related to particle size of both fat globules and protein, which affect their light reflectance and scattering ability; the size of these molecules is strongly influenced by choice of unit operations and processing parameters used (e.g., homogenisation) [26,27]. The soy samples had higher b* values (yellow colour) compared with the other yogurts and these results are in agreement with the results reported by Mei, Feng and Li [28], where also the a* (red-green axis) values were negative for all the soy samples. Even though the soy yogurt appeared white to the human eye, the chromameter data showed quantifiable differences in the green and yellow colour components [28]. Indeed, the b* values for soy yogurts can be associated with specific varieties of soybeans which are of yellow colour [29].

### 3.4. Rheological Analysis 

#### 3.4.1. Uniaxial Compression Testing

The results obtained from uniaxial compression testing (Table 5) showed that the hemp yogurt had higher firmness, consistency, cohesiveness and index of viscosity than the other samples. These textural properties can be related to the presence of the agar and the rice starch ingredients in the product formulation. Agar is widely used in the food industry for its ability to form strong gels at low concentrations and is also readily incorporated into food formulations since it does not require cations to gel, meaning that any variations in the cation concentrations contributed by the other components used in the formulation does not affect the agar gel performance [30]. Furthermore, the results for firmness, consistency, cohesiveness and index of viscosity for soy-1, coconut and cashew yogurt samples were comparable to the dairy product, with no significant differences between samples. The addition of hydrocolloids, even at low concentrations, is known to strongly influence the rheological properties of these types of products, particularly their synergies when applied in combination [31]. No correlation between texture and protein content can be observed due to the low protein content of the plant-based yogurts (0.60–4.60 g/100 g) and, therefore, the inclusion of hydrocolloids most strongly influenced the textural properties of these products. 

#### 3.4.2. Viscosity

**Figure 1:**
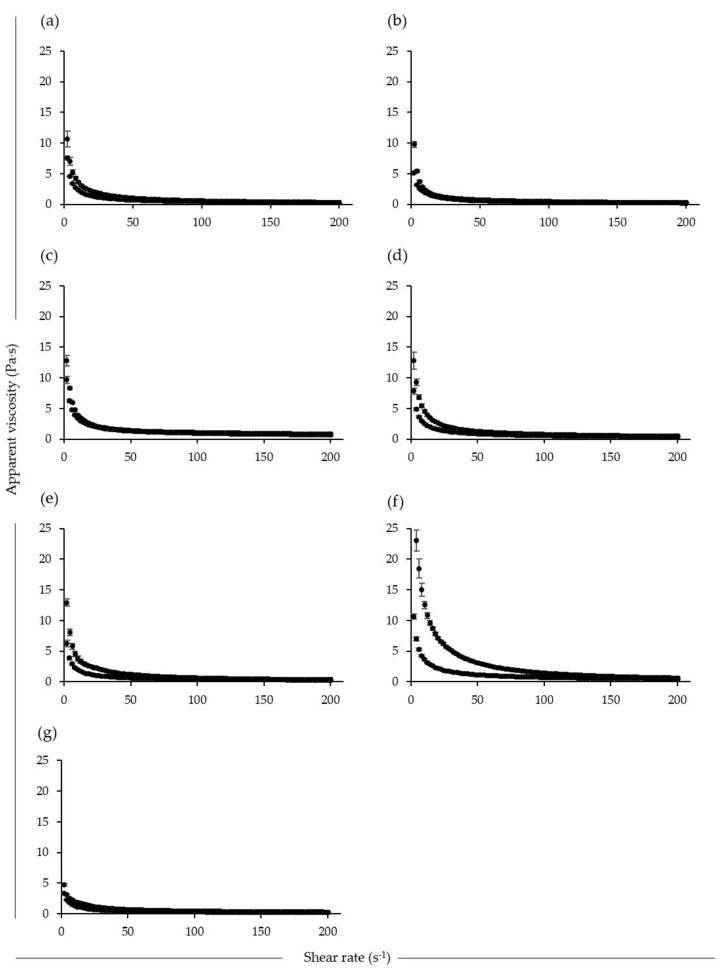
Apparent viscosity of soy-1 (**a**), soy-2 (**b**), coconut (**c**), cashew (**d**), almond (**e**), hemp (**f**), dairy (**g**) yogurts as a function of increasing shear rate from 0 to 200 s^−1^, on holding at 200 s^−1^ and on reducing shear rate from 200 to 0 s^−1^ at 20 °C.

Rheological parameters were calculated from measured raw data to describe the flow and shear-dependency behaviour of the yogurts (Table 6). The experimental data for the consistency coefficient (K) and flow behaviour index (n) fitted well to the Herschel-Bulkley model, with R^2^ values ranging from 0.80 to 1.00. Since the n values were all less than 1, the samples were described as having shear thinning flow behaviour. The K and n values of the soy yogurts were different to those reported by Donkor, Henriksson and Vasiljevic [32], where the authors reported values of 9.67 Pa·s^n^ and 0.21, respectively. The hemp product was an exception, where the Herschel-Bulkley model seemed not to be appropriate in describing the rheological flow behaviour of the yogurt. This sample also displayed a different apparent viscosity profile to the other products. The combination and interaction of rice starch and agar used in formulation of the hemp yogurt resulted in a strong impact on the rheological characteristics [33]. The K value of the dairy yogurt was statistically comparable to all othersamples, with the same trend observed for the n value. Moreover, the apparent viscosity profile for the soy-2 yogurt was similar to that for the dairy yogurt (Figure 1). The soy-2, coconut and dairy samples showed very little evidence of thixotropic behavior, while the soy-1, cashew and almond samples had clearly distinguishable thixotropic behavior, with the hemp sample being extremely thixotropic. The apparent viscosity profiles for both soy samples were in agreement with those for soy yogurts reported previously by Mei, Feng and Li [28]. The addition of gelling agents and hydrocolloids such as agar, starch, gums and/or pectin was found to significantly impact the rheological properties of the yogurt products. Combinations of these additives are frequently used in the food industry to obtain a desirable texture, either directly or through polysaccharide-protein interactions [34].

### 3.5. Water Holding Capacity

The water holding capacity analysis (Table 5) showed that the soy-2 yogurt was most similar to the dairy yogurt, with the latter showing results comparable to those reported by Singh and Muthukumarappan [35]. A possible explanation for this may be the absence of hydrocolloids in both products and the fact that the soy-2 yogurt had protein content similar to the dairy-based product. The physicochemical properties of soy protein are dependent on the protein profile components (i.e., β-conglycinin and glycinin), and also among the subunits of β-conglycinin or glycinin molecules [14]. The other plant-based yogurts containing hydrocolloids showed higher water holding capacity (>90%) compared to the dairy and soy-2 samples. 

### 3.6. Sensory Analysis 

The results for the sensory analysis were not significantly different for coconut, soy-1 and dairy yogurts (Table 7), following the same trend as the texture analysis data (Section 3.4.1). Indeed, the soy-1 yogurt had higher scores for texture (6.49), being statistically comparable to the dairy (6.33) and coconut (6.37) products. The dairy yogurt had the highest value (7.17) for the appearance attribute, while the result was not significantly different from the coconut (6.93) and soy-1 (6.82) samples. Moreover, the coconut yogurt showed the highest result for odour (6.43) and the acceptability was the highest (5.95) for both dairy and soy-1 samples, with the coconut yogurt being next most similar (5.19), with no significant differences. The panel were also asked to express their preference between the samples and 36% of the panelists preferred the dairy yogurt, while coconut and soy-1 samples were both preferred by 28% of the panel. The rest of the preferences (8%) were equally distributed between cashew and soy-2 yogurts, while the almond yogurt was not preferred by any of the panelists. 

In the questionnaire completed at the end of the test, information on panelist habits relating to yogurt consumption was requested and it was found that 92% of the panelists consumed dairy yogurt (36% everyday, 48% one to three times a week and 8% once a month), while only 32% consumed plant-based yogurts (4% one to three times a week, 12% once a month, 16% less than once a month). Furthermore, the food neophobia score showed that 44% of the panelists stated that they would change their diet for ethical/environmental reasons, 64% were constantly sampling new and different foods and 84% were not afraid to eat products never eaten before.

## 4. Conclusions

Selected chemical, rheological and sensory properties of six commercial plant-based yogurts and a dairy yogurt were studied. The results showed that selected plant-based yogurts had similar texture and sensory properties to the dairy-based product. For example, the uniaxial compression testing showed that the soy, coconut and cashew yogurt products were broadly comparable to the dairy product, attributing this to the use of hydrocolloids in the plant-based yogurts. In addition, some important textural attributes for yogurts, such as apparent viscosity and water holding capacity were similar between the dairy and selected plant-based yogurts, particularly the soy sample, which also had most similar protein content to the dairy yogurt. In the same way, the sensory analysis showed that the soy and coconut yogurts were appreciated just like the dairy-based product. The results obtained in this study allowed identification of key quality attributes of plant-based yogurts and highlighted important inter-relationships between such attributes and formulation which can be exploited in future product development.

## Figures and Tables

**Table 1:** Nutritional composition of plant-based yogurts (per 100 g of product).

	Unit	Soy-1	Soy-2	Coconut	Cashew	Almond	Hemp	Dairy
**Energy**	kJ/kcal	212/50	192/46	328/79	287/70	400/97	160/38	259/61
**Fat** ***of which saturated***	g g	2.30 0.40	2.60 0.40	4.90 4.20	4.20 0.80	7.90 0.70	2.00 0.20	1.50 1.00
**Carbohydrate** ***of which sugar***	g g	2.10 2.10	1.00 0.40	8.00 4.30	3.00 1.00	3.00 0.80	4.00 0.60	6.10 6.10
**Fibre**	g	1.00	0.10	0.20	n.a.	n.a.	n.a.	n.a.
**Protein**	g	4.00	4.60	0.60	2.00	2.30	0.60	5.10
**Salt**	g	0.25	0.07	0.40	0.10	0.36	0.03	0.18

n.a. = not available.

**Table 2:** List of ingredients used in the formulation of yogurts.

Product	Ingredients
**Soy-1**	Water, hulled soya beans (7.9%), sugar, calcium (tri-calcium citrate), stabiliser (pectin), acidity regulators (sodium citrate, citric acid), flavouring, sea salt, antioxidants (tocopherol-rich extract, ascorbyl palmitate), yogurt cultures (*S. thermophilus*, *L. bulgaricus*), vitamins (B12, D2)
**Soy-2**	Water, hulled soya bean (9%), calcium phosphate, bacterial cultures
**Coconut**	Water, coconut cream (20%), modified maize starch, dextrose, salt, thickener (pectin), colour (carotene), calcium phosphate, vitamin D2, vitamin B12, natural flavouring, non-dairy yogurt culture (*S. thermophilus*, *L. bulgaricus*)
**Cashew**	Organic cashew milk (97%) (organic cashews/filtered water), organic tapioca starch, organic carob gum, live vegan cultures
**Almond**	Almond milk (95%) (almonds, filtered water), tapioca starch, carob gum (thickener), live vegan cultures
**Hemp**	Hemp juice 96% (water, hemp seed 3%), rice starch, thickener (agar agar), selected live cultures of which *L. bifidus* and *L. acidophilus*, antioxidant (rosemary extract)
**Dairy**	Low fat milk, cultures

**Table 3:** Total titratable acidity (TTA), pH, D- and L-lactic acid content of yogurts.

Product	TTA (mL NaOH/g)	pH (-)	D-Lactic Acid g/100 g	L-Lactic Acid g/100 g
**Soy-1**	0.78 ± 0.01 ^b,c^	4.38 ± 0.00 ^b^	0.00 ± 0.00 ^a^	0.42 ± 0.01 ^b,c^
**Soy-2**	0.56 ± 0.01 ^a,b,c^	4.56 ± 0.00 ^b^	0.01 ± 0.00 ^a^	0.43 ± 0.01 ^b,c^
**Coconut**	0.49 ± 0.01 ^a,b,c^	4.00 ± 0.01 ^a^	0.01 ± 0.01 ^a^	0.36 ± 0.01 ^a,b,c^
**Cashew**	0.45 ± 0.01 ^a,b^	4.16 ± 0.01 ^a,b^	0.08 ± 0.00 ^a^	0.28 ± 0.01 ^a,b^
**Almond**	0.48 ± 0.01 ^a,b,c^	4.28 ± 0.01 ^a,b^	0.05 ± 0.00 ^a^	0.29 ± 0.00 ^a,b,c^
**Hemp**	0.12 ± 0.01 ^a^	3.99 ± 0.00 ^a^	0.01 ± 0.00 ^a^	0.10 ± 0.00 ^a^
**Dairy**	1.38 ± 0.02 ^c^	4.15 ± 0.01 ^a,b^	0.02 ± 0.00 ^a^	1.11 ± 0.01 ^c^

Values followed by different superscript letters (a–c) in the same column are significantly different (*p* < 0.05).

**Table 4:** Colour space values of yogurts.

Product	L*	a*	b*
**Soy-1**	64.2 ± 0.03 ^b,c,d^	−2.83 ± 0.01 ^a,b^	9.69 ± 0.01 ^d^
**Soy-2**	64.1 ± 0.01 ^b,c,d^	−1.62 ± 0.01 ^b,c,d^	7.95 ± 0.00 ^c,d^
**Coconut**	62.3 ± 0.41 ^a,b,c^	−1.79 ± 0.03 ^a,b,c^	4.31 ± 0.08 ^a,b^
**Cashew**	60.5 ± 0.11 ^a,b^	−0.98 ± 0.02 ^d^	6.85 ± 0.02 ^b,c,d^
**Almond**	64.2 ± 0.24 ^c,d^	−1.15 ± 0.01 ^c,d^	4.97 ± 0.04 ^a,b,c^
**Hemp**	60.2 ± 0.25 ^a^	−1.70 ± 0.01 ^a,b,c,d^	3.75 ± 0.03 ^a^
**Dairy**	66.6 ± 0.11 ^d^	−3.49 ± 0.02 ^a^	6.59 ± 0.02 ^a,b,c,d^

Values followed by different superscript letters (a–d) in the same column are significantly different (*p* < 0.05).

**Table 5:** Firmness, consistency, cohesiveness, index of viscosity and water holding capacity (*WHC*) of yogurts.

Product	Firmness (N)	Consistency (N·s)	Cohesiveness (N)	Index of Viscosity (N·s)	WHC (%)
**Soy-1**	0.46 ± 0.03 ^a,b,c^	9.98 ± 0.97 ^a,b,c^	0.28 ± 0.02 ^b,c^	5.63 ± 0.74 ^b,c^	96.3 ± 1.40 ^b,c,d^
**Soy-2**	0.73 ± 0.16 ^c,d^	14.1 ± 2.40 ^b,c,d^	0.39 ± 0.12 ^a,b^	7.76 ± 2.16 ^a,b^	82.8 ± 0.92 ^a,b^
**Coconut**	0.44 ± 0.02 ^a,b,c^	10.0 ± 0.23 ^a,b,c^	0.31 ± 0.02 ^a,b,c^	5.36 ± 0.10 ^b,c^	99.3 ± 0.50 ^d^
**Cashew**	0.51 ± 0.15 ^a,b^	8.71 ± 1.61 ^a,b^	0.27 ± 0.07 ^b,c^	5.33 ± 1.49 ^b,c^	97.2 ± 1.34 ^c,d^
**Almond**	0.72 ± 0.04 ^b,c,d^	15.1 ± 0.63 ^c,d^	0.44 ± 0.01 ^a,b^	9.26 ± 0.29 ^a,b^	91.0 ± 0.54 ^a,b,c^
**Hemp**	1.78 ± 0.03 ^d^	31.7 ± 2.53 ^d^	1.06 ± 0.03 ^a^	16.7 ± 0.66 ^a^	95.9 ± 2.31 ^b,c,d^
**Dairy**	0.36 ± 0.03 ^a^	6.81 ± 1.21 ^a^	0.23 ± 0.01 ^c^	3.61 ± 0.62 ^c^	75.7 ± 0.68 ^a^

Values followed by different superscript letters (a–d) in the same column are significantly different (*p* < 0.05).

**Table 6:** Apparent viscosity at 200 s^−1^, yield stress, consistency coefficient (K) and flow behaviour index (n) of yogurts.

Product	Yield Stress (Pa)	Apparent Viscosity at 200 s^−1^ (Pa·s)	K (Pa·s^n^)	*n* (-)	*R* ^2^
**Soy-1**	27.2 ± 2.50 ^a,b,c^	0.29 ± 0.01 ^a,b,c^	3.52 ± 0.06 ^b^	0.45 ± 0.00 ^a^	0.86 ± 0.01
**Soy-2**	20.9 ± 1.30 ^a,b^	0.23 ± 0.00 ^a^	0.77 ± 0.65 ^a^	0.72 ± 0.04 ^b^	0.94 ± 0.01
**Coconut**	30.4 ± 0.46 ^b,c^	0.75 ± 0.01 ^d^	1.34 ± 0.26 ^a^	0.87 ± 0.04 ^b^	1.00 ± 0.00
**Cashew**	35.6 ± 2.37 ^c^	0.42 ± 0.01 ^b,c,d^	2.75 ± 0.43 ^a,b^	0.58 ± 0.03 ^a,b^	0.97 ± 0.01
**Almond**	28.4 ± 1.94 ^b,c^	0.31 ± 0.02 ^a,b,c,d^	6.45 ± 1.62 ^b^	0.37 ± 0.05 ^a^	0.80 ± 0.03
**Hemp**	n.a.	0.55 ± 0.02 ^c,d^	n.a.	n.a.	n.a.
**Dairy**	11.7 ± 0.39 ^a^	0.24 ± 0.00 ^a,b^	2.50 ± 0.37 ^a,b^	0.55 ± 0.03 ^a,b^	0.91 ± 0.04

Values followed by different superscript letters (a–d) in the same column are significantly different (*p* < 0.05). n.a. = not applicable as the model doesn’t fit with the hemp sample.

**Table 7:** Hedonic test results for sensory evaluation of the yogurts.

	Soy-1	Soy-2	Coconut	Cashew	Almond	Dairy
**Appearance**	6.82 ± 0.01 ^c,d^	4.81 ± 1.20 ^a^	6.93 ± 0.30 ^c,d^	5.46 ± 0.39 ^a,b^	6.21 ± 0.36 ^b,c^	7.17 ± 0.18 ^d^
**Odour**	6.29 ± 0.05 ^c^	4.03 ± 0.49 ^a^	6.43 ± 0.35 ^c^	4.61 ± 0.64 ^a,b^	5.09 ± 0.46 ^b^	6.33 ± 0.18 ^c^
**Flavour**	5.75 ± 0.21 ^b^	2.54 ± 0.04 ^a^	4.79 ± 0.16 ^c^	2.60 ± 0.01 ^a^	2.88 ± 0.56 ^a^	5.67 ± 0.18 ^b,c^
**Texture**	6.49 ± 0.31 ^c^	4.05 ± 0.81 ^a^	6.37 ± 0.23 ^c^	5.17 ± 0.10 ^b^	4.83 ± 0.71 ^a,b^	6.33 ± 0.12 ^c^
**Acceptability**	5.95 ± 0.21 ^b^	2.80 ± 0.33 ^a^	5.19 ± 0.27 ^b^	3.61 ± 0.06 ^a^	3.54 ± 0.50 ^a^	5.95 ± 0.16 ^b^

Values followed by different superscript letters (a–d) in the same column are significantly different (*p* < 0.05).
